# Living with tardive dyskinesia: understanding the patient experience

**DOI:** 10.1017/S1092852926100856

**Published:** 2026-03-02

**Authors:** Leslie Citrome

**Affiliations:** Department of Psychiatry and Behavioral Sciences, New York Medical College, Valhalla, USA

**Keywords:** Functioning, impact, lived experience, patient experience, perception, tardive dyskinesia

## Abstract

After a brief overview of tardive dyskinesia, this targeted narrative review will examine the psychosocial consequences of tardive dyskinesia on patients’ daily lives, including stigma, social withdrawal, and quality of life, as well as impact on physical functioning. The extant literature on the impact of tardive dyskinesia on patients and their caregivers is described and summarized, including how patients with tardive dyskinesia perceive the severity and impact of their motor symptoms and whether this aligns with clinical observations by their treaters.

## Introduction

Tardive dyskinesia (TD), as defined in the 5th edition (text revised) version of the American Psychiatric Association’s Diagnostic and Statistical Manual of Mental Disorders, is “abnormal, involuntary movements of the tongue, jaw, trunk, or extremities that develop in association with the use of medications that block postsynaptic dopamine receptors, such as first- and second-generation antipsychotic medications and other medications such as metoclopramide for gastrointestinal disorders.”^1^ The description further adds, “In many cases, tardive dyskinesia is objectively mild, but, although it has been thought of as a cosmetic problem, it can be associated with significant distress and social avoidance. In severe cases, it may be associated with medical complications (eg, ulcers in cheeks and tongue; loss of teeth; macroglossia; difficulty in walking, swallowing, or breathing; muffled speech; weight loss; depression; suicidal ideation).”^1^ Thus, the impact of TD can go well beyond the observed stigmata of dyskinetic movements. TD is generally irreversible, but can be managed with medication treatment.

Although it is generally believed that second-generation antipsychotics (SGAs) are less likely to cause TD than first-generation antipsychotics (FGAs), the risk continues to be significant.^2^ ^–^^4^ Additionally, the indications for some SGAs have expanded to include bipolar disorder, major depressive disorder (adjunctive use), irritability associated with autistic disorder, Tourette’s Disorder, and agitation associated with dementia due to Alzheimer’s disease.^5^ In a meta-analysis published in 2017, the prevalence of TD was found to be 30% of patients receiving FGAs, 21% of patients receiving SGAs, and 7% in FGA-naive patients receiving SGAs.^2^ Thus, given the common occurrence of TD, it behooves health care providers to correctly identify the disorder,^6^ ^,^^7^ initiate appropriate treatment,^8^ and assess both the movements and the impact these movements have on the individual and their caregivers.^9^

The aim of this targeted narrative review is to describe the psychosocial consequences of tardive dyskinesia on patients’ daily lives, including stigma, social withdrawal, and quality of life, as well as impact on physical functioning.

## Methods

A search of the National Library of Medicine’s PubMed resource on the terms “tardive,” “dyskinesia,” and “impact” on December 7, 2025, and without any date or language constraints, resulted in 151 entries, of which 17 were relevant to the aims of this report. An additional search on the terms “tardive,” “dyskinesia,” and “perception” yielded 37 entries, of which an additional 3 reports were relevant. Papers that reported exclusively on monetary cost and/or resource utilization were excluded. A total of 16 publications were found that reported on the impact and/or perception of people with TD, their caregivers, and their providers, as assessed by surveys or consensus panels, as well as reports on data from retrospective and prospective studies.^9^ ^–^^24^ An additional 4 publications describe rating scales that can be used to assess the impact of TD.^25^ ^–^^28^ With the exception of 3 studies,^16^ ^,^^22^ ^,^^24^ all the studies were conducted or sponsored by one of the two pharmaceutical companies that manufacture treatments for TD,^29^ ^,^^30^ of which both are vesicular monoamine transporter 2 (VMAT2) inhibitors (valbenazine and deutetrabenazine), and both initially approved by the US Food and Drug Administration (FDA) in 2017.^8^

## Results

### Retrospective and prospective studies

The first mention of perception of TD and relationship to impact was in 1982.^24^ A total of 153 outpatients receiving antipsychotics were evaluated for TD using the Abnormal Involuntary Movement Scale (AIMS), with severity defined as the highest individual body area score (AIMS items 1 to 7), quantified in the range of 0–4 (none, minimal, mild, moderate, and severe), and representing the global judgment of severity of abnormal movements. Of the 70 patients with TD, 33% had minimal symptoms, 23% had mild symptoms, and 23% had moderate symptoms. None of the patients had severe TD. About one-third of the patients with TD were aware of their abnormal movements, with their awareness increasing with increased severity of their symptoms (the presence or absence of awareness was assessed using the global judgment of the patient’s reported awareness of abnormal movements). Of the 23 patients who were aware of their TD, 6 were moderately distressed, 10 were mildly distressed, and 7 were not distressed (the severity of distress was quantified by the global judgment of the patient’s reported awareness of abnormal movements). In a factor analysis, clinicians were most influenced by the severity of the patient’s oral dyskinetic movements when making a global judgment of severity. However, patients seemed aware of their TD and distressed by it when they experienced functional impairment, usually when their extremities were affected, as assessed by the global judgment of incapacitation due to abnormal movements. This item is notably devoid of any specific guidance regarding specific areas of incapacitation when assessing functional impact. The authors concluded that a significant number of patients may be aware of and distressed by their TD, and that this should be considered in treatment planning.

About 25 years after the above study, a cross-sectional study was conducted from 2005 to 2008 in Singapore that included 903 patients with schizophrenia and examined the impact of drug-induced Parkinsonism (DIP) and TD on Health-Related Quality of Life (HRQoL).^16^ Patients were assessed using the Simpson-Angus Scale, AIMS, and the EuroQoL five-dimensional utility scores (as algorithmically derived from the Positive and Negative Syndrome Scale). A total of 160 (17.7%) participants had only DIP, 119 (13.2%) had only TD, and 123 (13.6%) had both DIP and TD. HRQoL was lowest for patients with both DIP and TD, followed by the DIP only group, then the TD only group, and highest (best) in the group with neither TD nor DIP. Presence of TD only was not statistically significantly associated with HRQoL.

After FDA approval of the VMAT2 inhibitors for the treatment of TD in 2017, a number of additional studies were conducted. Cumulative burden of illness was examined in US veterans with TD and schizophrenia/schizoaffective, or bipolar or major depressive disorders, and who were receiving antipsychotics from October 2014 through September 2015.^15^ Among 7985 veterans, 332 (4.2%) were diagnosed as having possible TD. Veterans with TD had a higher Charlson Comorbidity Index score and higher odds of any medical hospitalization. There was no association between the Charlson Comorbidity Index and total AIMS score within the TD group after adjusting for age, sex, and schizophrenia. The authors concluded that TD “may be a marker for patients at risk of adverse health care outcomes and diminished quality of life.”

Burden of TD in long-term care settings was examined retrospectively using claims data from 2294 patients receiving care through commercial insurance, Medicaid, or Medicare, with a TD diagnosis code, who had at least one long-term care stay, and had sufficient continuous claims data during the period from January 2016 through December 2022.^20^ A high comorbidity burden was observed on the Mean Charlson Comorbidity Index. Almost one-half of the population received ≥3 medications with central nervous system properties. However, there were no comparisons between this group and an equivalent group without TD.

A prospective real-world screening study (RE-KINECT) was conducted from 2017 to 2019 and included 739 patients from 37 outpatient psychiatry clinics in the United States.^31^ The study included adults with ≥3 months of lifetime antipsychotic exposure and ≥1 psychiatric disorder. Two cohorts were examined: patients with no abnormal involuntary movements or whose movements were not consistent with possible TD based on clinician assessment, and patients with clinician-confirmed possible TD. The latter group was subdivided into two: those who also self-reported having abnormal involuntary movements and those who did not. Assessments included the EQ-5D-5L assessing health, the Sheehan Disability Scale (SDS) assessing social functioning, and patient- and clinician-rated severity of possible TD, as well as patient-rated impact of possible TD. RE-KINECT has so far resulted in 3 publications.^12^ ^–^^14^ As reported, 204 of the participants (27.6%) had clinician-confirmed possible TD.^12^ EQ-5D-5L score was significantly worse in patients with TD than in those without TD.^12^ In the 110 patients with TD who were aware of their TD, patient-rated impact was significantly associated with EQ-5D-5L and SDS scoring.^13^ Patient-rated severity was also significantly associated with the EQ-5D-5L. However, the association between clinician-rated severity with the EQ-5D-5L and SDS was not statistically significant. The authors concluded that clinician-rated severity of TD may not always correlate with patient perceptions of the significance of TD. AS part of the RE-KINECT study, caregivers who were unpaid were assessed by questionnaire.^14^ Based on responses from the 36 caregivers who noticed patients’ TD and who were caring for individuals who also noticed their TD, caregiver ratings of the movements were significantly correlated with patient ratings (but not with clinician ratings). About half of the caregivers reported impairment in their own ability to continue usual activities, be productive, socialize, or take care of themselves, because of the patient’s TD.

In sum, retrospective and prospective studies have documented impairment of TD in HRQoL, functioning, and associated measures, apart from the movements themselves. Information about impact from the caregiver can be especially important as their perspectives generally align with those of the patient, in contrast to the discordance that can be observed between patient and provider assessment of this impact.

### Patient social media and online surveys

**Figure 1.**
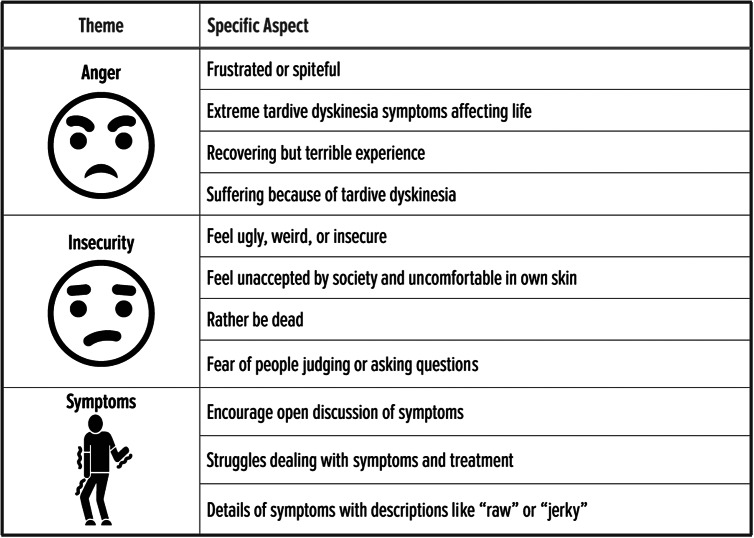
Theme analysis used in Farrar and colleagues. Reproduced from Fig. 5 in Farrar et al.,^10^ as per the Creative Commons Attribution 4.0 International License, http://creativecommons.org/licenses/by/4.0/.

The FDA has encouraged the collection of comprehensive and representative input from patients, including the use of social media tools.^32^ Social Media Listening (SML) is one of the accepted techniques and has been used to describe patient experiences for a variety of conditions, as evidenced by 33 PubMed entries containing the text “social media listening” in the title of each publication (ascertained as of December 10, 2025). Farrar and colleagues report on an SML study where English-language online content from 2017 to 2019 on social media platforms, blogs, and forums was analyzed regarding patient or caregiver experiences of TD, from a total of 261 postings.^10^ Posts were primarily from forums (47%) and Twitter (33%), and revealed that 64% of the content was consistent with negative valence, with patients often feeling angry about having TD from a medication used to treat a different condition, and being frustrated about the negative impact of their TD, as well as feeling insecure (including feeling ugly, weird, or self-conscious), feeling unaccepted by society, and fearing of being judged by others. The thematic framework used in this report is shown in Figure 1. These observations are entirely consistent with this writer’s experience conducting Instagram question and answer sessions with social media influencers in 2022–2023 as part of a general public campaign on TD organized by Med-IQ (http://med-iq.com).

A cross-sectional web-based survey of adult psychiatric patients with clinician-confirmed diagnosis of TD (N = 197) or clinician-confirmed absence of TD (N = 219) was conducted in the United States in 2017, the year that FDA approved the two treatments available for TD.^11^ Patients had an underlying clinician-confirmed diagnosis of bipolar disorder (N = 143), major depressive disorder (N = 140), or schizophrenia (N = 133), distributed almost equally between the 2 study groups. The survey was structured and utilized the SF-12v2, a 12-item questionnaire measuring functioning and well-being, the Quality of Life Enjoyment and Satisfaction Questionnaire Short Form (Q-LES-Q-SF), a 16-item survey, Health-Related Quality of Life (HRQoL), and the Social Withdrawal subscale of the Internalized Stigma of Mental Illness scale (SW-ISMI), a 6-item short form of the 29-item ISMI survey, measuring exacerbation of mental illness stigma on social functioning due to TD. Across all participants with bipolar disorder, major depressive disorder, and schizophrenia, patients without a TD diagnosis had significantly better HRQoL and less social withdrawal than patients with TD.

A survey was conducted online in the US in 2020–2021 among adults with TD.^17^ Participants with self-reported schizophrenia (N = 74), bipolar disorder (N = 134), or major depressive disorder (N = 61), rated the impact of TD on their physical (eg, dressing, eating, sleep, speech, other daily activities), psychological (eg, mood, irritability, energy level, anxiety, concentration, motivation, embarrassment, shyness, self-esteem, fear of rejection, memory), and social functioning (recreational activities, social activities, taking public transport, shopping, leaving the house) over the past week using a scale from 1–5, with higher scores describing greater impact. A questionnaire was also completed regarding work productivity and activity impairment, and participants also described the impact that TD had on their underlying psychiatric condition. Mean impact scores were 3.1 for physical consequences, 3.5 for psychological consequences, and 3.2 for social consequences, with higher scores for those with greater severity of their TD, and the highest scores for those with schizophrenia. However, participants with mild or moderate TD rated their impact similarly, with mean scores of 2.6, 3.1, and 2.8, for the physical, psychological, and social domains, respectively. Two-thirds of participants reported activity impairment because of TD. Participants who were employed (N = 193) reported rates of about 29% for absenteeism, 68% for presenteeism (impairment while working), and 74% with overall work impairment. About one-third to one-half of participants reported skipping, reducing the dose, or stopping their antipsychotic medication, and stopping visits to their providers because of TD. One-fifth of participants advised someone else not to take an antipsychotic medication. Coping strategies for patients with TD included slowing down when speaking, massage/stretch/shake body parts that are cramping/moving, muscle relaxant medication, sleeping pills, covering hands, getting up and walking when movements start, meditation/breathing exercises, sucking on hard candy or chewing gum, covering their face, and postponing activity.

In another online survey conducted in 2022, data from 435 patients from Australia, Brazil, China, and South Korea were examined (3 patients from Israel were excluded because, as per the authors, the number of participants from that country was too low).^21^ Patients self-reported a physician diagnosis of TD and schizophrenia, schizoaffective disorder, bipolar disorder, or major depressive disorder, and exhibited ‘extra, irregular movements’. Although only about a third of patients reported that their TD symptoms were severe or very severe, about 85% of all patients reported a moderate to high impact of their TD symptoms on quality of life, with substantial negative impacts of TD on their daily lives, emotional and psychological states, and social activities. About one-quarter of patients reported that TD often/always affected their treatment adherence for their underlying psychiatric condition, or that TD worsened their underlying psychiatric condition. About one-half reported that they developed other psychiatric conditions. Although there was heterogeneity in the reported type of impact, results were generally consistent with what was found in the United States.^17^

A broader cross-sectional survey conducted online in 2022 aimed to assess the tolerability of antipsychotic medication side effects for patients with schizophrenia (N = 200).^22^ Extrapyramidal side effects such as pseudoparkinsonism and tardive dyskinesia, as well as feeling detached and memory and cognitive issues, were ranked as the least tolerable. Among the 100 caregivers also surveyed, this ranking remained essentially the same.

Although the number of participants in the above SML study and surveys were relatively small, and represented patients active on social media or willing to participate in an online survey, the sentiments expressed reveal substantial self-reported impact of their TD on several dimensions of their lives, often beyond the phenomenology of the dyskinetic movements themselves, as reflected in quantifiable impact even in the presence of mild or moderate TD severity. Moreover, it was observed that TD can also affect medication adherence and how patients manage their underlying condition.

### Caregiver surveys

An online survey was conducted in the United States among 162 caregivers.^18^ Caregivers were defined as unpaid family members or friends who were providing care for ≥3 months to a patient with diagnoses of TD and schizophrenia, bipolar disorder, and/or major depressive disorder. Similar to the patient survey described above,^17^ the impact of TD on the patient’s physical and social functioning was assessed over the past week using a scale from 1 to 5. Additionally, the impact of TD on these domains in the caregivers’ own personal and professional lives was also enumerated. About four-fifths of caregivers reported that TD had a severe impact on the cared-for patients’ physical, psychological, and social functioning. Over three-quarters of caregivers reported that TD sometimes/often/always impacted the ability of the patients to socialize or leave the house. Over half of caregivers reported that others sometimes/often/always stared or looked at the patient and inquired about the abnormal movements.

Mean impact scores for the caregivers themselves were 2.1 for TD-related caregiving tasks, 2.5 for caregiver psychological well-being, and 2.7 for caregiver daily activities, each domain score increasing with TD symptom severity. For caregivers of patients with mild to moderate symptoms, scores were 2.0, 2.3, and 2.6, respectively, and were similar to those observed for caregivers of patients with severe to very severe TD symptoms, where scores were 2.4, 2.7, and 2.9, respectively. Half of caregivers reported impairment in regular activities such as housework or shopping. Overall, about one-quarter of caregivers reported a severe impact of TD on their own lives. Among the 136 caregivers who were employed, about half experienced overall work impairment because of TD-related caregiving.

Pragmatically, two-thirds to three-quarters of caregivers provided assistance for shopping for groceries, preparing meals, managing medications, doing household chores, and driving a car. The most burdensome task reported was assistance with bathing or showering. Over one-third reported feeling anxious or worried because of the patient’s TD. Other common negative emotions included feeling sad or unhappy, overwhelmed, overburdened, and stressed or strained. About a third of caregivers reported an impact on their ability to enjoy the things they do for fun, to join social activities, to socialize with friends, and/or to date or meet new people.

Although the number of caregivers in the survey was relatively small and represented people willing to participate in an online survey, the self-reported impact of caring for people with TD was substantial.

### General population surveys

Attitudes about people with TD were assessed in a blinded digital survey where a total of 4800 people were recruited.^23^ Participants were randomized 1:1 to a test or control group to view a video of a professional actor simulating TD movements or no TD movements prior to completing surveys on employment, dating, and friendship domains. For the employment and dating domains, screening criteria were used to ensure that participants in the employment survey were required to have hiring/interviewing experience, and participants in the dating survey were required to be single and open to online/blind dates in the next 6 months. Separate samples of participants were used when evaluating mild-to-moderate or moderate-to-severe TD movements (ie, 2400 participants each), and further divided so that participants would be tested in only one domain. In all domains, participants responded significantly less favorably to persons with TD movements, whether the movements were mild-to-moderate or moderate-to-severe. Fewer participants considered the candidate with TD as a potential employee (29.2% and 22.7% fewer, for mild-to-moderate and moderate-to-severe TD, respectively), found him/her attractive (20.5% and 18.7% fewer), or interested in becoming friends with him/her (12.3% and 16.5% fewer).

### Provider consensus panel and surveys

In 2021, an advisory panel of 7 experts in psychiatry and movement disorder neurology published recommendations on the assessment of the impact of TD.^9^ After literature review and two virtual consensus panel meetings conducted in 2020, consensus was reached that “as part of routine clinical practice, it is imperative to assess the impact of TD on the patient’s life to help guide treatment decisions.” Five categories of domains were articulated: social, physical, vocational, and psychological functioning, and the impact of TD on the underlying psychiatric disorder. Moreover, in addition to receiving input from patients, caregivers and family members should also be involved in assessing impact. The point was made that although patients can present with what is considered to be “mild” TD based on assessment of abnormal movements, even mild dyskinetic movements can cause social anxiety and potential impairments in eating, speaking, breathing, and ambulation. As such, there was consensus that the subjective term “mild” should be avoided when making a diagnosis of TD.

An online survey of 163 clinicians (physicians, nurse practitioners, and physician assistants) who prescribed valbenazine for TD within the past 24 months, reported outcomes from medical records of their patients with TD who were being treated with VMAT2 inhibitors (N = 601).^19^ Prior to treatment, 93% of the patients showed impairment in ≥1 social domain, and 88% were impaired in ≥ 1 physical domain. After treatment, among those with improvement in TD symptoms (N = 540), 80%–95% showed improvement in social domains, 90%–95% showed improvement in physical domains, and 73% showed improvement in their underlying psychiatric condition. The authors concluded that, as part of the routine process of treating TD, the impact of TD should be assessed at the same time as when the dyskinetic movements themselves are evaluated.

In the same project reported earlier that surveyed patients,^21^ 340 physicians from Australia, Brazil, China, South Korea, and Israel also participated in the online survey. Physician participants were neurologists, neuropsychiatrists, or psychiatrists who had been in practice for ≥3 years, spent ≥60% of their time in direct patient care, and had treated ≥3 patients with TD in the previous 2 years. Their responses were not linked to the patient responses. About 88% of physicians reported a moderate to high impact of TD symptoms on quality of life for their patients, a number similar to what was reported by patients (85%). Physicians also reported substantial negative impacts of TD on patients’ daily lives, emotional and psychological states, and social activities. Physicians also reported that TD affected adherence (75%), worsened the underlying psychiatric condition (71%), and led to the development of other psychiatric conditions (78%); these percentages were higher than what patients themselves reported (29%, 25%, and 47%, respectively). Other differences between the reports from providers and patients, as identified, included that physicians reported physical and emotional/psychological/social effects of TD as being almost equally impactful, whereas patients generally reported a greater impact of emotional/psychological/social functioning than physical functioning.

To summarize, expert consensus encourages the assessment of the impact of TD on a routine basis. The need for this is additionally reinforced by provider surveys that demonstrate the presence of substantial impairment, with some differences from what was reported by the patients themselves.

### Rating scales

It is clear from the above that clinicians’ observation of TD movements alone as mild, moderate, or severe is not necessarily consistent with the patients’ experienced impact, particularly when so-called mild TD is associated with marked distress. Tools have been created to help ascertain the type and severity of TD impact. The AIMS itself contains two global measures that help address this (incapacitation due to abnormal movements, and patient’s awareness and related distress), but there is insufficient guidance as to what should be asked of the patient—the instructions for the AIMS simply note “Ask patient whether he/she notices any movements in mouth, face, hands, or feet. If yes, ask to describe and to what extent they currently bother patient or interfere with his/her activities.”^33^

The Clinician’s Tardive Inventory (CTI), available for download at https://ars.els-cdn.com/content/image/1-s2.0-S135380202500553X-mmc1.docx, is a new scale developed for clinicians to better describe the clinical signs and functional impact of TD.^25^ ^,^^26^ The scale was devised by a movement disorder neurologist and further refined by a committee of neurologists and psychiatrists. In addition to rating a plethora of abnormal movements, functional impairments scored include activities of daily living (ADL), leisure or occupation, social impairment, symptom distress, and physical harm, each rated 0–3 with 0 = patient is unaware or unaffected, 1 = symptoms mildly impact patient, 2 = symptoms moderately impact patient, and 3 = symptoms severely impact patient. A total functional impairment score ranges from 0 to 3, with 0 = patient is unaware or unaffected, to 3 = symptoms severely impact the patient, and is calculated as the maximum impact score among the domains of social impairment, ADL, symptom distress, and physical harm. In addition, specific individual movements, for example, tongue/mouth movements, are reported alongside a checklist of related patient-reported functional impairments, such as slurred speech, difficulty eating/drinking/swallowing saliva, weight loss, inability to wear dentures, dry mouth, tongue or mouth sores, pain, social embarrassment, occupational impairment, and impairment of non-occupational daily activities.

In an effort to create a clinician rating scale focused solely on impact, a modified Delphi process was used to assess agreement on the format and content of the Impact-TD scale^27^ (available for download at https://www.austedohcp.com/globalassets/austedohcp/impact-td-scale.pdf). Experts involved included movement disorder neurologists and psychiatrists. Consensus was reached on the categorization of 4 functional domains: social, psychological/psychiatric, physical, and vocational/educational/recreational, with each domain scored from 0 (no impact) to 3 (severe impact), and with a global Impact-TD score based on the highest single score for any domain. Lists of suggested queries are provided for each domain. For example, for the social domain, queries include “difficulty participating in events with family and others (eg, holiday gatherings, religious institution attendance); self-consciousness/embarrassment about movements or being seen/asked about by others (eg, stigma, rejection); avoidance of interaction with others (eg, declines invitations, avoids leaving home, isolation); reduced quality of interpersonal communication (eg, distraction from conversation, problems interpreting body language).”

A patient-reported outcome measure focused on impact has also been developed^28^ called the Tardive Dyskinesia Impact Scale (TDIS). The instrument was primarily based on patient and caregiver input. This scale is available upon request from https://www.neurocrinemedical.com/tdis-request-form/. Items were chosen based on the most common and bothersome impacts using questions free of medical jargon, and capture the physical and socio-emotional impacts of TD. Psychometric properties of the scale were assessed using clinical trial data of valbenazine for the treatment of TD. Six domains (mouth/throat function, dexterity, mobility, pain, social, and emotional) are assessed across 11 items (speech, mouth noises, swallowing, gripping, writing, walking, balance, leg pain, unwanted attention, embarrassment, and self-consciousness). Each item is scored from 0 (not at all or never) to 4 (extremely or all the time).

In summary, regarding scales that address impact, the AIMS is limited to 2 items without elaboration, and although the CTI is intended to be a comprehensive TD scale that includes a description of both the movements and the impact of the movements, it is unlikely to replace the AIMS, given the ubiquity of the AIMS in the clinic^7^ and in the conduct of clinical trials.^8^ The Impact-TD scale and the TDIS are intended to be companions to movement-focused scales such as the AIMS, with the Impact-TD scored by a clinician and the TDIS by the patient. Use of these instruments can better describe the patient experience living with TD.

## Discussion

In the 3rd edition of the American Psychiatric Association Practice Guideline for the Treatment of Patients With Schizophrenia, it is recommended that patients who have moderate to severe or disabling tardive dyskinesia associated with antipsychotic therapy be treated with a VMAT2 inhibitor.^34^ Moreover, the guidance adds that treatment with a VMAT2 inhibitor can also be considered for patients with mild TD based on patient preference, associated impairment, or effect on psychosocial functioning. The inclusion of language such as *disabling*, *impairment*, and *psychosocial functioning* addresses the notion that TD goes beyond the movements themselves, and thus a severity rating solely based on the frequency and amplitude of the dyskinetic movements would be inadequate when assessing a patient with TD. As evidenced in retrospective and prospective studies, and surveys of patients, caregivers, providers, and the general population, living with TD means living with the physical, psychiatric, and psychosocial consequences of the disorder, as well as resulting in additional caregiver burden. There is an incongruence between patient and provider assessments of severity, as severity is in the “eye of the beholder,” and the impact of TD is not necessarily dependent solely on the objective severity of TD movements.

In the consensus report on the screening, diagnosis, and treatment of TD cited earlier,^6^ consensus was reached that a patient with 1 rating of mild severity (≥ 2 on AIMS) affecting 1 body area should be considered as having possible TD. When appraising this, if the movement causes embarrassment and social isolation, or impairment working in a public-facing occupation, or other consequences, the rating of the TD as “mild” would not be consistent with the patient’s own assessment of the disorder. The consensus panel on the impact of TD^9^ reaffirmed that the impact of TD is not necessarily dependent solely on the objective severity of TD movements. The report further adds that the impact of TD on mental health and wellness could worsen existing psychiatric comorbidities. Physical limitations may prevent the patient with TD from performing the motor skills we all take for granted, such as eating, speaking, swallowing, handling objects, and ambulation. TD movements can be stigmatizing, and for the general public who does not know what TD is, may assume that the afflicted individual is under the influence of street drugs or alcohol. Thus, the impact of TD on day-to-day life is of importance and should be routinely assessed by health care providers.

Although the use of rating scales can be helpful, they can be burdensome in terms of time. It does not appear that the rather lengthy CTI will displace the AIMS as the standard for assessing dyskinetic movements, even though it does a far better job at assessing the functional impact of the myriad of abnormal movements associated with TD. Patient-reported outcome scales such as the TDIS may be more easily adopted in routine practice in the treatment of movement disorders (the analogy of the patient-rated PHQ-9 for the assessment of depression comes to mind). However, the Impact-TD scale does include useful prompts for questions that can easily be incorporated in the standard dialogue one employs when assessing patients with TD.

## Conclusion

Living with TD means not only living with abnormal involuntary movements that are generally irreversible, but also coping with varying impairments in social, psychological/psychiatric, physical, and vocational/educational/recreational functioning. Caregiver burden can also be substantial. Clinicians need to be mindful that only assessing the frequency and amplitude of the abnormal movements omits the essential driver of treatment—the patient’s lived experience with the disease.

## CNS spectrums

### CME review article

#### Living with tardive dyskinesia: understanding the patient experience

*This CME activity is provided by HMP Education and Neuroscience Education Institute (NEI).*

#### CME/CE information

**Target Audience:** This activity has been developed for the healthcare team or individual prescriber specializing in mental health. All other healthcare team members interested in psychopharmacology are welcome for advanced study.

**Learning Objectives:** After completing this educational activity, you should be better able to:

- Examine the psychosocial consequences of tardive dyskinesia on patients’ daily lives, including stigma, social withdrawal, and quality of life.
- Evaluate how patients perceive the severity and impact of their TD symptoms and whether this aligns with clinical observations.
- Discuss strategies to improve patient outcomes by addressing the physical, emotional, and social challenges posed by TD.

**Accreditation:** In support of improving patient care, this activity has been planned and implemented by HMP Education and Neuroscience Education Institute (NEI). HMP Education is jointly accredited by the Accreditation Council for Continuing Medical Education (ACCME), the Accreditation Council for Pharmacy Education (ACPE), and the American Nurses Credentialing Center (ANCC) to provide continuing education for the healthcare team.

**Activity Overview:** This activity is best supported via a computer or device with current versions of the following browsers: Mozilla Firefox, Google Chrome, or Safari. A PDF reader is required for print publications. A post-test score of 70% or higher is required to receive CME/CE credit.

**Estimated Time to Complete:** 1 hour.

Continuing Education credit will be available for three (3) years from the publication date of the associated article. Please visit https://nei.global/cnsspectrums2025 for additional information and to access the CE activity.

**NEI maintains a record of participation for six (6) years.*

**Instructions for Optional Posttest and CME Credit**

1. Read the article
2. Successfully complete the posttest at https://nei.global/CNS/TD-02
3. Print your certificate

Questions? Email customerservice@neiglobal.com.

**Credit Designations:** The following are being offered for this activity:

- **Physician: ACCME *AMA PRA Category 1 Credits*™**
  - HMP Education designates this enduring material for a maximum of 1.00 *AMA PRA Category 1 Credit*™. Physicians should claim only the credit commensurate with the extent of their participation in the activity.
- **Nurse: ANCC contact hours**
  - This continuing nursing education activity awards 1.00 contact hour. Provider approved by the California Board of Registered Nursing, Provider #18006 for 1.00 contact hour.
- **Nurse Practitioner: ACCME *AMA PRA Category 1 Credit*™**
  - American Academy of Nurse Practitioners National Certification Program accepts *AMA PRA Category 1 Credits*™ from organizations accredited by the ACCME.
  - The content in this activity pertaining to pharmacology is worth 1.00 continuing education hours of pharmacotherapeutics.
- **Pharmacy: ACPE application-based contact hours**
  - This internet-based, knowledge-based activity has been approved for a maximum of 1.00 contact hour (.10 CEU).
  - *The official record of credit will be in the CPE Monitor system. Following ACPE Policy, NEI and HMP Education must transmit your claim to CPE Monitor within 60 days from the date you complete this CPE activity and are unable to report your claimed credit after this 60-day period. Ensure your profile includes your DOB and NABP ID.*
- **Physician Associate/Assistant: AAPA Category 1 CME credits**
  - HMP Education has been authorized by the American Academy of PAs (AAPA) to award AAPA Category 1 CME credits for activities planned in accordance with the AAPA CME Criteria. This internet enduring activity is designated for 1.00 AAPA Category 1 credit. Approval is valid until February 11, 2028. PAs should only claim credit commensurate with the extent of their participation.
- **Psychology: APA CE credits**
  - Continuing Education (CE) credits for psychologists are provided through the co-sponsorship of the American Psychological Association (APA) Office of Continuing Education in Psychology (CEP). The APA CEP Office maintains responsibility for the content of the programs. This activity awards 1.00 CE Credit.
- **Social Work: ASWB-ACE CE credits**
  - As a Jointly Accredited Organization, HMP Education is approved to offer social work continuing education by the Association of Social Work Boards (ASWB) Approved Continuing Education (ACE) program. Organizations, not individual courses, are approved under this program. Regulatory boards are the final authority on courses accepted for continuing education credit. Social workers completing this internet enduring course receive 1.00 general continuing education credit.
- **Non-Physician Member of the Healthcare Team: Certificate of Participation**
  - HMP Education awards hours of participation (consistent with the designated number of *AMA PRA Category 1 Credit*™) to a participant who successfully completes this educational activity.

**Peer Review:** The content was peer-reviewed by an MD, MPH specializing in forensics, psychosis, schizophrenia, mood disorders, anxiety, and cognitive disorders — to ensure the scientific accuracy and medical relevance of information presented and its independence from commercial bias. NEI and HMP Education takes responsibility for the content, quality, and scientific integrity of this CME/CE activity.

**Disclosures:** All individuals in a position to influence or control content are required to disclose any relevant financial relationships. Any relevant financial relationships were mitigated prior to the activity being planned, developed, or presented.
***Faculty Author/Presenter***

**Leslie Citrome, MD, MPH**
*Clinical Professor of Psychiatry and Behavioral Sciences, New York Medical College, Valhalla, NY* Consultant/Advisor: AbbVie/Allergan, Acadia, Adamas, Alkermes, Angelini, Astellas, Avanir, Axsome, Biogen, BioXcel, Bristol-Myers Squibb, Boehringer Ingelheim, Cadent Therapeutics, roCerevel, Clinilabs, COMPASS, Delpor, Eisai, Enteris BioPharma, HLS Therapeutics, Idorsia, INmune Bio, Impel, Intra-Cellular Therapies, Janssen, Karuna, Lundbeck, Luye, Lyndra, MapLight, Marvin, Medavante-ProPhase, Merck, Mitsubishi-Tanabe Pharma, Neumora, Neurocrine, Neurelis, Noema, Novartis, Noven, Otsuka, Ovid, Praxis, Recordati, Relmada, Reviva, Sage, Sumitomo/Sunovion, Supernus, Teva, University of Arizona, Vanda, Wells Fargo, and ad hoc consulting for marketing, commercial, or scientific scoping research Speakers Bureau: AbbVie/Allergan, Acadia, Alkermes, Angelini, Axsome, BioXcel, Bristol-Myers Squibb, Eisai, Idorsia, Intra-Cellular Therapies, Janssen, Lundbeck, Neurocrine,f Noven, Otsuka, Recordati, Sage, Sunovion, Takeda, Teva, Vanda, and CME activities organized by Medscape, NACCME, NEI, Vindico, and Universities/Professional Organizations Stockholder: AbbVie/Allergan, Acadia, Alkermes, Angelini, Axsome, BioXcel, Bristol-Myers Squibb, Eisai, Idorsia, Intra-Cellular Therapies, Janssen, Lundbeck, Neurocrine,f Noven, Otsuka, Recordati, Sage, Sunovion, Takeda, Teva, Vanda, and CME activities organized by Medscape, NACCME, NEI, Vindico, and Universities/Professional Organizations Stock Options: Reviva Royalties/Publishing: Taylor & Francis (Editor-in-Chief, Current Medical Research and Opinion), Wiley (Editor-in-Chief, International Journal of Clinical Practice, through 2019), UpToDate (Reviewer), Springer Healthcare (Book), Elsevier (Topic Editor, Psychiatry, Clinical Therapeutics).

The remaining Planning Committee members, Content Editors, Peer Reviewers, and NEI planners/staff have no financial relationships to disclose. NEI and HMP Education planners and staff include Gabriela Alarcón, PhD, Caroline O’Brien, MS, Ali Holladay, Andrea Zimmerman, EdD, CHCP, Samantha Joy, Antonio Aldridge, and Steven S. Simring, MD, MPH.

**Disclosure of Off-Label Use:** This educational activity may include discussion of unlabeled and/or investigational uses of agents that are not currently labeled for such use by the FDA. Please consult the product prescribing information for full disclosure of labeled uses.

**Cultural Linguistic Competency and Implicit Bias:** A variety of resources addressing cultural and linguistic competencies and strategies for understanding and reducing implicit bias can be found in this handout—download me.

**Accessibility Statement.**

For questions regarding this educational activity, or to cancel your account, please email customerservice@neiglobal.com.

**Support:** This activity is supported by an unrestricted educational grant from Neurocrine Biosciences.
